# Epigenetic dysregulation in mycosis fungoides and sézary syndrome

**DOI:** 10.3389/fcell.2026.1813995

**Published:** 2026-05-11

**Authors:** Vinícius de Camargo Callefi, Isadora Alves, Nélio Cézar De Aquino, Sofia Cattena, Emanuelle Santos, Ketelyn Vasconcelos, Hebert Fabricio Culler, Luís Alberto de Pádua Covas Lage, Vanderson Rocha, Adriana Castello Costa Girardi, José Antonio Sanches, Carlos Alejandro Murga-Zamalloa, Juliana Pereira, Cadiele Oliana Reichert

**Affiliations:** 1 Real-World Evidence Observatory and Lab for Precision Public Health, Laboratory of Medical Investigation in Pathogenesis and Directed Therapy in Onco-Immuno-Hematology (LIM-31), Department of Hematology, Hemotherapy and Cell Therapy - Faculty of Medicine, University of São Paulo (FM-USP), São Paulo, Brazil; 2 Laboratory of Medical Investigation in Pathogenesis and Directed Therapy in Onco-Immuno-Hematology (LIM-31), Department of Hematology, Hemotherapy and Cell Therapy - Faculty of Medicine, University of São Paulo (FM-USP), São Paulo, Brazil; 3 Brazilian Health Regulatory Agency (Agência Nacional de Vigilância Sanitária - ANVISA), Brasília, Brazil; 4 Department of Pharmaceutical Assistance and Strategic Inputs (DAF), Ministry of Health, Esplanada dos Ministérios, Brasília, Brazil; 5 Cancer Institute of the State of São Paulo (ICESP) Octávio Frias de Oliveira, São Paulo, Brazil; 6 Laboratory of Renal Physiology and Cardiometabolism, Department of Cardiopneumology, University of São Paulo (FMUSP), São Paulo, Brazil; 7 Department of Dermatology, University of São Paulo Medical School, São Paulo, Brazil; 8 Department of Pathology, University of Illinois at Chicago, Chicago, IL, United States

**Keywords:** cutaneous T-cell lymphoma (CTCL), DNA methylation, epigenetics, HDAC inhibitors, histone acetylation, microRNA, precision medicine, therapeutic resistance

## Abstract

Cutaneous T-cell lymphomas (CTCL), including mycosis fungoides (MF) and Sézary syndrome (SS), remain challenging to diagnose at early stages and to manage durably. Although staging and classification have improved, early MF can mimic benign inflammatory dermatoses, and advanced disease frequently develops resistance to standard therapies, including histone deacetylase (HDAC) inhibitors. Here, we summarize how aberrant DNA methylation and histone modifications contribute to CTCL pathogenesis, promote a permissive tumor microenvironment, and enable immune escape, as well as emerging epigenetic biomarkers, such as microRNA signatures and promoter hypermethylation, that may improve diagnostic accuracy and patient stratification. Finally, we discuss resistance mechanisms linked to epigenetic plasticity and intratumoral heterogeneity and highlight future strategies combining rational drug regimens with longitudinal single-cell profiling to improve the durability of response.

## 1 Introduction

Mycosis fungoides (MF) and Sézary syndrome (SS) are categorized as cutaneous T-cell lymphomas (CTCL), a subset of non-Hodgkin lymphomas involving primarily the skin (Willemze, 2022; Kempf et al., 2024; Hristov et al., 2025). Within CTCL, MF is identified as the predominant subtype, accounting for almost 60% of cases. In contrast, SS is a rare and aggressive leukemic variant, with a reported frequency of 2% and a poor 5-year disease-specific survival of only 36% according to the World Health Organization–European Organization for Research and Treatment of Cancer (WHO–EORTC) data (Willemze, 2022; Zhang and Zhang, 2021). MF and SS are characterized by clinical variability, ranging from indolent, skin-limited disease to advanced-stage disease that may involve extracutaneous compartments, including lymph nodes, peripheral blood, or visceral sites, such as liver, spleen and bone marrow (Willemze, 2022; Hristov et al., 2025). Given that primary cutaneous lymphomas are described as rare, heterogeneous, and extranodal malignancies, their classification and treatment are based on the integration of clinical information, histopathologic assessment, and the TNMB staging system. Clinicopathological and genetic-molecular correlations are also emphasized as important when a definite diagnosis is sought (Willemze, 2022; Hristov et al., 2025).

However, histology alone may be insufficient for CTCL classification, and MF, mainly at the patch or plaque stage, is described as diagnostically difficult due to overlap with reactive processes. Additionally, SS skin biopsies may be subtle, non-specific and can resemble MF (Willemze, 2022; Hristov et al., 2025). As a consequence, delayed diagnosis is common, with many patients experiencing years of symptoms before a definitive diagnosis is established (Hristov et al., 2025; García-Díaz et al., 2021). These diagnostic limitations highlight the importance of integrating complementary clinical, histopathologic, and genetic-molecular approaches, and have motivated the development of novel diagnostic tools (Willemze, 2022; García-Díaz et al., 2021). Moreover, clinical presentation and behavior may correlate with histologic, immunophenotypic, and genetic features (Willemze, 2022).

To refine this diagnostic process, high-throughput sequencing of the T-cell receptor can evaluate tumor clone frequency in the skin. This metric offers independent prognostic value regarding progression-free and overall survival outcomes for patients with early-stage disease (de Masson et al., 2018). Furthermore, clinical risk stratification has been strengthened by the Cutaneous Lymphoma International Prognostic Index (CLIPI). Validated within the PROCLIPI cohort, this index successfully stratifies patients with advanced-stage disease into distinct prognostic risk groups, providing a more precise estimate of survival probabilities (Bashir et al., 2025; Scarisbrick et al., 2025).

Despite the multiple therapies recommended by the National Comprehensive Cancer Network (NCCN), including systemic therapies such as histone deacetylase inhibitors (HDACi) vorinostat and romidepsin (Zhang and Zhang, 2021; Olsen et al., 2007), and conventional cytotoxic chemotherapy, which often induces only short-term remissions (Hristov et al., 2025), curative options remain a subject of evolving consensus. Although autologous stem-cell transplantation (ASCT) was previously considered a potentially curative option in CTCL, the most recent evidence-based guidelines from the American Society for Transplantation and Cellular Therapy (ASTCT) and the United States Cutaneous Lymphoma Consortium (USCLC) now explicitly recommend against the use of ASCT for MF/SS outside of clinical trials (Zhang and Zhang, 2021; Goyal et al., 2024).

Instead, allogeneic hematopoietic stem cell transplantation (allo-HSCT) is established as a potentially curative treatment modality for advanced disease (Hristov et al., 2025; Goyal et al., 2024). In the prospective CUTALLO study, allogeneic HSCT resulted in longer progression-free survival than non-HSCT treatment in patients with advanced-stage CTCL, a finding that supports the clinical rationale for a graft-versus-lymphoma effect (de Masson et al., 2023). Driven by these clinical outcomes, the 2025 consensus guidelines from the European Society for Blood and Marrow Transplantation (EBMT) formally advocate for allo-HSCT in eligible individuals with advanced-stage MF or SS who exhibit disease progression or lack of response after a minimum of two prior systemic regimens (Damaj et al., 2025). Furthermore, this updated framework emphasizes the necessity of early transplant evaluation to optimize patient care (Damaj et al., 2025).

HDACi are associated with relatively low overall response rates in CTCL, generally around 30%–40%, with outcomes varying across agents and studies (Zhang and Zhang, 2021; García-Díaz et al., 2021). CTCL subtypes, including MF and SS, demonstrate extensive heterogeneity, and relapsed/refractory (R/R) disease is discussed in the therapeutic context. Recent studies describe diverse genetic and epigenetic alterations, including changes involving chromatin modification. Single-cell sequencing studies have revealed substantial heterogeneity among malignant T cells in CTCL, while cytogenetic and genomic studies have provided evidence of apoptosis resistance mechanisms and molecular diversity across malignant T-cell populations (Tensen et al., 2022; Yamagishi, 2022; Du et al., 2025).

While genomic landscapes in CTCL are characterized by extensive heterogeneity, genetic alterations alone do not fully account for disease pathogenesis. This is particularly evident in MF, where sequencing studies have highlighted fewer recurrent alterations than in SS, in which copy-number abnormalities and driver mutations involving genes such as TP53 and PLCG1 are more consistently observed (Tensen et al., 2022; Mirza et al., 2020). Consequently, the dysregulation of key gene-expression programs likely depends, at least in part, on epigenomic control (Yamagishi, 2022).

The genetic framework of these lymphomas includes a range of somatic mutations and copy number variations (Fléchon et al., 2024). Sequencing data reveals repeated defects in genes connected to T-cell receptor and NF-κB pathways, prominently PLCG1 and TNFAIP3, as well as alterations within the JAK/STAT signaling cascade (Fléchon et al., 2024; Choi et al., 2015). These genomic events routinely appear alongside abnormalities in epigenetic regulators and chromatin remodeling, suggesting that the disease’s development involves a combined contribution of genetic alterations and epigenomic disruptions (Fléchon et al., 2024; Choi et al., 2015).

In addition to isolated recurrent mutations, the genomic architecture of CTCL is marked by pronounced interpatient and intrapatient heterogeneity, including copy-number alterations, subclonal evolution, and pathway-level convergence involving T-cell receptor/NF-κB signaling, JAK/STAT activation, cell-cycle control, DNA-damage response, and chromatin regulation. In MF, recent genomic profiling studies further suggest that clonality patterns and the acquisition of additional genomic events may help identify patients at higher risk of progression independently of clinicopathologic stage. These findings support the view that CTCL biology is best interpreted through an integrated genomic-epigenomic framework rather than through single-gene abnormalities alone (Tensen et al., 2022; Fléchon et al., 2024).

**FIGURE 1:**
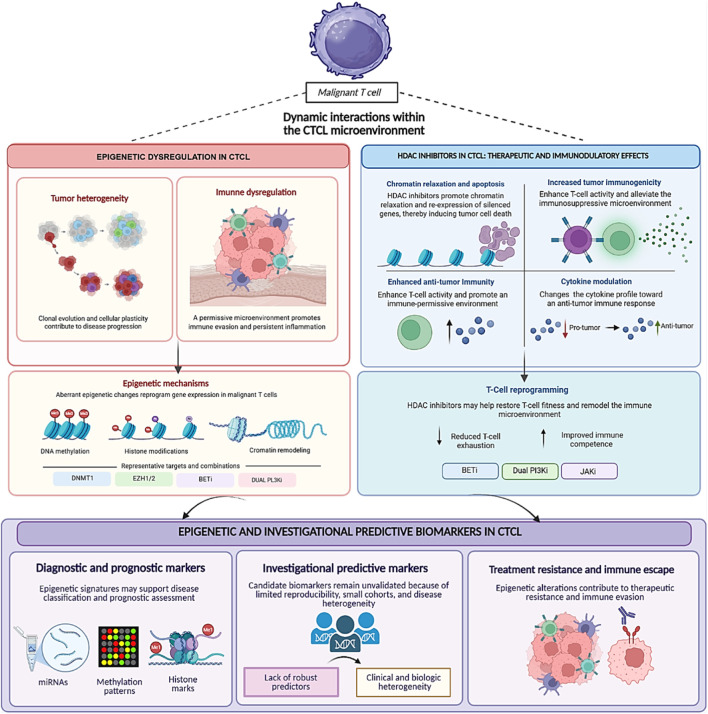
Schematic overview of epigenetic dysregulation in cutaneous T-cell lymphoma (CTCL). DNA methylation abnormalities, histone-modifying alterations, chromatin remodeling, and microRNA dysregulation contribute to tumor heterogeneity, immune dysregulation, and disease progression in mycosis fungoides and sézary syndrome. The figure also summarizes the therapeutic and immunomodulatory effects of HDAC inhibitors and highlights the investigational status of predictive biomarkers of treatment response in CTCL.

Epigenetic regulators, primarily DNA methylation and histone modifications, orchestrate chromatin accessibility to precisely dictate gene expression levels and timing. These mechanisms are fundamental to cellular differentiation and developmental processes (Yamagishi, 2022; Hara and Sawada, 2022; Tao et al., 2024). Evidence in MF and SS implicates epigenetic alterations in malignant transformation and disease progression and highlights their relevance to treatment resistance, as epigenetic changes can modulate therapeutic sensitivity and have been linked to resistance to HDAC inhibitors (Zhang and Zhang, 2021; Hara and Sawada, 2022; Tao et al., 2024). This mini-review summarizes recent evidence on epigenetic mechanisms underlying diagnostic challenges, biomarker development, and therapeutic resistance in mycosis fungoides and Sézary syndrome (Figure 1).

## 2 Epigenetic dysregulation in mycosis fungoides and sézary syndrome

The epigenetic landscape of CTCL, including MF and SS, involves coordinated alterations in DNA methylation and histone regulation that reshape chromatin structure and gene expression programs, thereby contributing to malignant transformation and disease progression (Zhang and Zhang, 2021; Hara and Sawada, 2022). A central mechanism described in CTCL is the imbalance between global DNA hypomethylation, which may promote genomic instability, and focal hypermethylation of CpG-rich promoter regions, which can silence tumor suppressor genes (Zhang and Zhang, 2021; Hara and Sawada, 2022). In this context, promoter hypermethylation of CDKN2A has been associated with impaired cell-cycle control, while additional epigenetic silencing events involving CDKN2B and MGMT have also been reported in CTCL subsets (Zhang and Zhang, 2021; Tensen et al., 2022; Hara and Sawada, 2022). Together, these abnormalities cooperate with histone-based regulatory changes to sustain the malignant phenotype (Zhang and Zhang, 2021; Hara and Sawada, 2022).

In parallel with DNA methylation changes, dysregulation of histone modifiers also plays a pivotal role in CTCL pathogenesis. The histone methyltransferase EZH2 is frequently overexpressed in aggressive CTCL and is associated with increased levels of the repressive chromatin mark H3K27me3, contributing to transcriptional silencing of genes involved in differentiation and immune regulation (Tensen et al., 2022; Yamagishi, 2022; Isshiki et al., 2025; Sneeringer et al., 2010). As alterations in histone acetylation are also prominent, HDAC1, HDAC2, and HDAC6 have been reported to be overexpressed in CTCL, creating a chromatin environment that favors transcriptional repression of tumor suppressor pathways (Zhang and Zhang, 2021; García-Díaz et al., 2021). Together, these epigenetic abnormalities help sustain malignant-cell survival and may contribute to therapeutic resistance (Hara and Sawada, 2022; Tao et al., 2024; Wu et al., 2021).

## 3 Epigenetic biomarkers for diagnosis and prognostic stratification

To optimize precision medicine in CTCL, it is necessary to systematically distinguish between diagnostic, prognostic, and predictive biomarkers. Diagnostic biomarkers, encompassing specific molecular drivers and epigenetic signatures, are important for unequivocally distinguishing early-stage malignant T-cell clones from benign inflammatory dermatoses (Zhang et al., 2026). In contrast, prognostic biomarkers provide critical information regarding the intrinsic aggressiveness and likely clinical course of the disease, irrespective of the therapeutic regimen applied; for instance, high-throughput sequencing and comprehensive genomic profiling have been established as robust prognostic tools to identify subclonal heterogeneity and predict rapid disease progression (de Masson et al., 2018; Fléchon et al., 2024).

Finally, predictive biomarkers are intended to estimate the likelihood that a patient will respond to a specific therapeutic intervention. In CTCL, baseline chromatin accessibility patterns have shown potential to predict response to histone deacetylase inhibitors in research settings (Qu et al., 2017). These data suggest that pre-treatment epigenetic states may influence therapeutic sensitivity, but the evidence remains investigational and has not yet translated into a clinically validated predictive biomarker for routine use in MF/SS (Qu et al., 2017).

The clinical management of MF and SS is profoundly impacted by diagnostic delays, with patients often presenting a history of non-specific skin lesions for several years, sometimes exceeding a decade, before a definitive diagnosis is reached (García-Díaz et al., 2021). This latency is primarily due to the great imitator nature of early-stage MF, which clinically and histologically mimics benign inflammatory dermatoses (BID) such as chronic dermatitis, lichen planus, or psoriasis (Willemze, 2022; Kempf et al., 2024). According to the WHO–EORTC guidelines, while the gold standard remains the clinico-pathological correlation, histology alone frequently fails to distinguish malignant T-cell infiltrates from reactive ones in early patches, where cytologic atypia may be minimal (Willemze, 2022; Kempf et al., 2024).

To overcome these morphological limitations, epigenetic profiling has emerged as a promising diagnostic adjunct. In particular, microRNA expression patterns provide high-precision molecular signatures for CTCL classification. A diagnostic classifier based on differential expression of miR-155, miR-203, and miR-205 was established and shown to distinguish CTCL from benign inflammatory dermatoses with high accuracy (Ralfkiaer et al., 2011). In addition, miR-155 overexpression has been associated with disease progression and malignant-cell survival in MF and SS (Tensen et al., 2022; Ralfkiaer et al., 2011; Moyal et al., 2017).

Although microRNA-based classifiers have clear diagnostic relevance in CTCL, this progress has not yet translated into routine clinical implementation, and microRNA-directed therapies remain investigational. In particular, miR-155 inhibition has shown biological rationale and encouraging preclinical activity, but these approaches have not yet resulted in an established therapeutic strategy for MF and SS in routine practice. Therefore, at present, microRNAs are more mature as candidate diagnostic biomarkers than as clinically established therapeutic tools (Ralfkiaer et al., 2011; Seto et al., 2018). Thus, although microRNA signatures may support CTCL diagnosis, microRNA-directed therapies have not yet translated into a practice-changing therapeutic advance in MF/SS.

DNA methylation analysis provides an additional layer of diagnostic support. Aberrant promoter hypermethylation of genes such as CDKN2A and CDKN2B has been associated with malignant CTCL phenotypes and is not typically observed in reactive inflammatory dermatoses, supporting its value as a candidate biomarker in challenging cases (Tensen et al., 2022; Hara and Sawada, 2022). In parallel, integration of epigenetic markers with next-generation sequencing approaches can improve detection of T-cell receptor clonality and related genomic abnormalities beyond conventional methods (de Masson et al., 2018; Tensen et al., 2022). In this setting, molecular tools should be regarded as complementary to clinicopathologic assessment, helping refine diagnostically challenging cases rather than replacing histology and immunophenotyping (Table 1).

**TABLE 1:** Epigenetic alterations in CTCL: diagnostic, prognostic, predictive, and therapeutic implications.

Alteration/Biomarker	Biomarker category	Epigenetic mechanism	Associated cellular phenotype	Translational relevance	Representative methods	References
Global DNA hypomethylation with focal promoter hypermethylation	Diagnostic/exploratory	DNA methylation dysregulation	Genome-wide hypomethylation with locus-specific hypermethylation may reprogram transcription and contribute to silencing of tumor-suppressor genes	Provides a conceptual framework for methylation-based biomarkers in CTCL, particularly when integrated with transcriptional and genomic data	DNA methylome profiling; promoter methylation assays; integrative methylome-transcriptome analyses	Zhang and Zhang (2021), Tensen et al. (2022)
CDKN2A (p16) promoter hypermethylation	Diagnostic/biologic stratification	DNA methylation	Epigenetic silencing of a cell-cycle regulator associated with impaired cell-cycle control; higher methylation has been reported in erythrodermic MF	Candidate biomarker of cell-cycle deregulation and disease biology; may help support molecular stratification in selected CTCL subsets	CDKN2A promoter methylation analysis; correlation with gene-expression readouts	Zhang and Zhang (2021), Tensen et al. (2022)
CDKN2B (p15) promoter hypermethylation	Diagnostic/exploratory	DNA methylation	Epigenetic silencing of a tumor suppressor involved in negative regulation of the cell cycle	Candidate biomarker of tumor-suppressor gene silencing in a subset of CTCL cases	CDKN2B promoter methylation analysis; integration with expression data where available	Zhang and Zhang (2021), Tensen et al. (2022)
MGMT promoter hypermethylation	Exploratory/biologic subgroup marker	DNA methylation	Reduced expression of a DNA-repair enzyme involved in removal of O6-alkylguanine lesions	Candidate epigenetic subgroup marker that may reflect altered DNA-repair capacity in a subset of CTCL	MGMT promoter methylation assessment in CTCL specimens; correlation with MGMT expression/function	Zhang and Zhang (2021), Tensen et al. (2022), Hara and Sawada (2022)
EZH2/H3K27me3	Therapeutic target/exploratory biomarker	Histone methylation and Polycomb repression	EZH2 overexpression and increased H3K27me3 contribute to transcriptional repression of genes involved in differentiation and immune regulation	Provides rationale for therapeutic targeting of PRC2/EZH2 and for monitoring H3K27 methylation as a downstream pharmacodynamic readout	IHC/IF for EZH2 and H3K27me3; chromatin mapping; perturbation studies with EZH1/2 inhibitors such as valemetostat	Isshiki et al. (2025), Yi et al. (2018), Teubner et al. (2026), Zinzani et al. (2024)
HDAC1/HDAC2/HDAC6 overexpression and activation	Therapeutic target/response-associated feature	Histone acetylation regulation	Increased HDAC activity contributes to a chromatin state that favors malignant-cell survival; HDAC6 activation via IL-15 signaling has been linked to survival pathways	Provides biological rationale for the clinical use of HDAC inhibitors and for development of rational combination strategies targeting resistance-associated pathways	IHC/IF and expression profiling for HDAC1/2/6; histone/protein acetylation assays; drug-sensitivity and resistance models	Zhang and Zhang (2021), Olsen et al. (2007), Zhu et al. (2024), Shih et al. (2024)
Baseline chromatin accessibility/epigenetic regulome patterns associated with HDAC inhibitor response	Predictive (investigational)	Chromatin accessibility and transcriptional state	Distinct baseline chromatin states may be associated with differential sensitivity or resistance to HDAC inhibitors	Promising predictive biomarker candidate for HDAC inhibitor response in research settings; not clinically validated for routine use in MF/SS	ATAC-seq; chromatin accessibility profiling; integrative epigenomic-transcriptomic analysis; correlative biomarker studies	Qu et al. (2017)
miR-155 upregulation	Prognostic/therapeutic target	Non-coding RNA regulation	miR-155 overexpression has been associated with progression from patch to plaque and tumor-stage lesions and may support malignant-cell proliferation/survival	Candidate biomarker associated with more advanced disease biology and a potential therapeutic target	miRNA profiling; RT-qPCR validation; anti-miR-155 perturbation studies in CTCL models; xenograft assays	Ralfkiaer et al. (2011), Moyal et al. (2017), Seto et al. (2018)
Diagnostic microRNA signature (miR-155 high, miR-203 low, miR-205 low)	Diagnostic	Non-coding RNA regulation	A specific microRNA pattern has been reported to distinguish CTCL from benign inflammatory dermatoses	Promising diagnostic classifier for CTCL versus benign inflammatory dermatoses; additional validation is needed before routine clinical implementation	miRNA profiling; microarray; RT-qPCR classifier panels; sensitivity/specificity analyses	Ralfkiaer et al. (2011)
Modest clinical responses to HDAC inhibitors and comparator data from MAVORIC	Response-associated clinical feature	Epigenetic therapy response	Approved HDAC inhibitors show modest ORRs in relapsed/refractory CTCL; mogamulizumab showed higher ORR than vorinostat in MAVORIC	Highlights the need for predictive biomarkers and rational combinations beyond HDAC inhibitor monotherapy	Correlative endpoints in HDAC inhibitor studies; response/resistance analyses; resistant-cell models	Zhang and Zhang (2021), Olsen et al. (2007), Kim et al. (2018), Whittaker et al. (2010)
PI3K inhibition combined with HDAC inhibition	Therapeutic strategy/response-modifying approach	Signaling-epigenetic crosstalk	Combined pathway blockade may enhance apoptosis and help overcome adaptive resistance to HDAC inhibitors	Supports further evaluation of combination strategies designed to improve efficacy and overcome resistance to HDAC inhibitor monotherapy	Drug-screening and synergy assays; patient-derived xenograft models; translational combination studies	Wu et al. (2021), Ford et al. (2025)
Single-cell and longitudinal profiling	Exploratory/biomarker discovery platform	Multi-omic profiling	Reveals malignant T-cell heterogeneity, resistant subclones, and dynamic cell states associated with progression and treatment response	Supports discovery of future cell-state–specific biomarkers and mechanisms of treatment response and resistance	Single-cell RNA-seq; paired TCR sequencing; longitudinal sampling	Tensen et al. (2022), Yamagishi (2022), Du et al. (2025), Srinivas et al. (2024), Chennareddy et al. (2025)

Unless otherwise specified, the epigenetic signatures, molecular targets, and response-associated profiles listed in this table should be considered candidate or investigational biomarkers. Although several show strong biological rationale and preclinical or translational support, none has yet demonstrated sufficient analytical validation, clinical validation, and real-world reproducibility to support routine prediction of treatment response in CTCL.

## 4 Epigenetic mechanisms of therapeutic resistance and immune escape

Despite the integration of epigenetic therapies into the therapeutic landscape, MF and SS remain chronic relapsing diseases in which long-term disease control is difficult to achieve, particularly in advanced stages (Hristov et al., 2025; García-Díaz et al., 2021). This challenge is illustrated by pivotal HDAC inhibitor trials. In the MAVORIC study, patients assigned to vorinostat had a median progression-free survival of 3.1 months, underscoring the limited durability of response with HDAC inhibitor monotherapy in relapsed/refractory CTCL (Kim et al., 2018). Likewise, long-term follow-up of romidepsin showed an overall response rate of approximately 34%, indicating that a substantial proportion of patients either do not respond adequately or fail to maintain response over time (Whittaker et al., 2010).

Even with the promise of targeted signaling inhibition, clinical outcomes remain heterogeneous. For instance, while brentuximab vedotin has demonstrated significant efficacy in CD30^+^ cases (Prince et al., 2017), and pembrolizumab shows activity in refractory disease (Khodadoust et al., 2020), the emergence of large cell transformation (LCT) continues to represent a formidable barrier, typically conferring a more aggressive clinical course and reduced sensitivity to standard regimens (Agar et al., 2010).

Mechanistic studies suggest that resistance to HDAC inhibitors may emerge through activation of compensatory survival pathways. For example, resistance to vorinostat has been linked to IL-2Ralpha-associated signaling, whereas resistance to romidepsin has been associated with persistent JAK/STAT pathway activation (Zhu et al., 2024; Shih et al., 2024). More broadly, current evidence supports a model in which epigenetic plasticity and intratumoral heterogeneity allow malignant cells to evade the effects of single-agent therapies by engaging alternative transcriptional and signaling programs (Tensen et al., 2022; Du et al., 2025; Srinivas et al., 2024; Deshpande and Munoz, 2022).

Although the biological rationale for targeting oncogenic signaling pathways in CTCL is strong, major clinical breakthroughs with pathway-directed agents have remained limited. This is particularly evident for JAK inhibition: despite recurrent evidence of JAK/STAT dysregulation in CTCL and case-based or early clinical signals of activity, the efficacy and safety of JAK inhibitors in MF/SS remain insufficiently defined in controlled studies (Packer and Brunner, 2025). These observations reinforce the need for rational combination approaches, particularly those integrating epigenetic therapies with signal-transduction blockade to address clonal heterogeneity and adaptive resistance (García-Díaz et al., 2021; Shih et al., 2024; Olsen et al., 2022). Accordingly, no major clinical breakthrough has yet been achieved with signaling-pathway targeting in CTCL, including JAK-directed approaches.

This concept has been further refined by single-cell sequencing studies, which show that CTCL represents a complex ecosystem in which minor subclones may display distinct transcriptional and chromatin states associated with resistance to apoptosis and therapeutic escape (Du et al., 2025; Srinivas et al., 2024; Chennareddy et al., 2025). Additionally, resistance extends to the host immune system; epigenetic reprogramming can drive T cells toward exhaustion, impairing anti-tumor immunity and rendering the tumor microenvironment permissive to neoplastic cell growth (Singh et al., 2024).

The cutaneous microbiome, particularly *Staphylococcus aureus*, also contributes to immune evasion and therapeutic resistance in CTCL. *Staphylococcus aureus* infection releases alpha-toxin, which can impair CD8^+^ T-cell–mediated cytotoxicity against tumor cells and thereby promote immune escape and disease progression (Blümel et al., 2020). In addition, enterotoxins from this pathogen can induce neoplastic T cells to secrete cytokines that compromise epidermal barrier integrity. This process occurs via the JAK-mediated downregulation of essential keratinocyte structural components (Gluud et al., 2026). Crucially, these microbial enterotoxins also promote cellular survival against conventional treatments, including the HDAC inhibitor romidepsin, by engaging TCR, NF-κB, and JAK/STAT signaling cascades within the malignant clone (Vadivel et al., 2024).

### 4.1 The immunomodulatory role of HDAC inhibitors

HDAC inhibitors exert direct cytotoxic and pro-apoptotic effects on malignant T-cell clones while also modulating the tumor microenvironment (Qu et al., 2017). Epigenetic modulation by these agents can effectively reprogram the immunological landscape by significantly enhancing the expression of major histocompatibility complex (MHC) class I and II molecules, as well as tumor-associated antigens (Zhang et al., 2026; Zhang et al., 2025). This increase in antigen presentation facilitates the recognition of neoplastic cells by the host’s immune system, thereby augmenting the intrinsic immunogenicity of the tumor and reversing the epigenetic immune evasion mechanisms characteristic of advanced CTCL (Qu et al., 2017; Zhang et al., 2025).

HDAC inhibition may also influence surrounding immune effector cells and help restore immunosurveillance. Recent evidence suggests that HDAC inhibitor treatment may reinvigorate exhausted CD8^+^ cytotoxic T cells and favorably modulate immunosuppressive regulatory T-cell (Treg) populations (Tian et al., 2025). These effects may also promote a more favorable cytokine profile and a more effective anti-tumor immune response, linking direct tumor cell killing to broader immune modulation (Qu et al., 2017).

Together, these findings support further evaluation of HDAC inhibitors in combination immunotherapy strategies and maintenance approaches (Tian et al., 2025). Response to HDAC inhibitors remains heterogeneous, and this biological complexity may partly explain why predictive biomarkers have been difficult to validate in routine practice. At present, no biomarker has demonstrated sufficient analytical validation, clinical validation, and real-world reproducibility to support routine prediction of HDAC inhibitor response in MF/SS, despite promising associations between baseline epigenetic regulomes and clinical outcomes (de Masson et al., 2018; Qu et al., 2017; Zhang et al., 2025).

## 5 Future perspectives

Recent literature suggests that CTCL management is evolving from a predominantly morphological framework toward a more molecularly informed and combinatorial approach (Zhang et al., 2026). Global registry data underscore the importance of precise clinical risk stratification to optimize treatment selection and improve outcomes in advanced-stage disease (Bashir et al., 2025; Scarisbrick et al., 2025). Concurrently, high-throughput T-cell receptor sequencing and comprehensive genomic profiling have increasingly revealed subclonal heterogeneity and identified patients at higher risk of disease progression, independently of clinicopathologic stage (de Masson et al., 2018; Fléchon et al., 2024). Recognition of the cutaneous microbiome, particularly the role of *S. aureus* in drug resistance and barrier dysfunction, further highlights the limitations of targeting only tumor-intrinsic pathways (Gluud et al., 2026; Vadivel et al., 2024). In refractory disease, durable control may depend in part on effective immunomodulatory strategies, as illustrated by the progression-free survival benefit observed with allogeneic stem cell transplantation (de Masson et al., 2023).

To ensure clinical consistency, the evaluation of disease burden and treatment response must strictly adhere to the updated consensus recommendations from the ISCL, USCLC, and EORTC. These guidelines prioritize ‘time to next treatment’ (TTNT) as a key clinical endpoint and emphasize integrated staging that accounts for blood, node, and visceral involvement alongside skin assessment (Olsen et al., 2022).

Building upon the necessity to overcome therapeutic resistance and sustain clinical remission, the continuous pharmacological targeting of the epigenome has emerged as a critical strategy. Mechanistically, HDACi exert their antineoplastic effects by dynamically remodeling the chromatin accessibility landscape; in responsive patients, this intervention progressively exposes repressed pro-apoptotic loci, such as the *FAS* gene, thereby restoring essential apoptotic pathways within the malignant T-cell clones (Qu et al., 2017).

Translating these epigenetic effects into durable clinical benefit has historically been challenging in advanced disease. However, the recent RESMAIN trial provided pivotal clinical evidence by establishing the HDAC inhibitor resminostat as an efficacious maintenance therapy. Importantly, these data indicate that the role of HDAC inhibition in CTCL should not be viewed exclusively in the context of induction or salvage therapy, as maintenance treatment may also provide clinically meaningful disease control in advanced-stage MF and SS (Stadler et al., 2026).

While epigenetic therapies represent an important advance in CTCL management, current single-agent approaches remain limited by the durability of response. A major unmet need is the identification of reliable biomarkers capable of predicting which patients are most likely to benefit from HDAC inhibitors before treatment initiation. Although molecular classifiers such as microRNA signatures have improved diagnostic stratification, predictive biomarkers for therapeutic efficacy remain lacking in routine clinical practice (Zhang and Zhang, 2021; Tensen et al., 2022; Ralfkiaer et al., 2011).

Given these limitations, the field is increasingly moving toward combinatorial strategies designed to induce synthetic lethality and overcome adaptive resistance. It has been demonstrated that PI3K inhibition can resensitize resistant CTCL cells to HDAC inhibitors, with synergistic antitumor effects observed in patient-derived xenograft models (Wu et al., 2021). Supporting this rationale, recent real-world evidence has also suggested clinical activity for combined PI3K and HDAC inhibition in relapsed/refractory T-cell lymphomas, including cutaneous forms (Ford et al., 2025).

The therapeutic landscape is also expanding to include upstream epigenetic regulators beyond HDACs. The overexpression of EZH2 in aggressive CTCL has positioned EZH1/2 dual inhibitors, such as valemetostat, as promising agents in T-cell lymphomas. The VALENTINE-PTCL01 study showed durable responses with valemetostat in relapsed or refractory peripheral T-cell lymphomas, while preclinical work suggests that EZH2 inhibition may also enhance lymphoma immunogenicity and improve T-cell function, supporting future combination strategies (Yamagishi, 2022; Isshiki et al., 2025; Teubner et al., 2026; Zinzani et al., 2024).

Finally, addressing intratumoral heterogeneity will require a shift from static bulk profiling to dynamic single-cell monitoring. Recent single-cell RNA sequencing studies have revealed subset-specific molecular phenotypes and substantial heterogeneity in CTCL gene-expression programs, reinforcing the importance of longitudinal profiling to track the emergence of resistant subclones under therapeutic pressure and to support more adaptive treatment strategies (Du et al., 2025; Srinivas et al., 2024; Chennareddy et al., 2025).

In conclusion, integration of epigenetic mechanisms into the clinical understanding of MF and SS has shifted the paradigm from viewing these diseases as static entities to recognizing them as dynamic and evolving ecosystems. While promoter hypermethylation and histone modifications offer valuable diagnostic biomarkers, they also orchestrate the transcriptional plasticity that fuels therapeutic resistance. The failure of single-agent HDAC inhibitors to provide durable responses underscores the need for combinatorial approaches that target multiple layers of the epigenome or exploit synthetic lethality pathways. Ultimately, the future of CTCL management lies in precision epigenetics, including the use of single-cell technologies to monitor clonal evolution and rational drug combinations to disrupt adaptive survival mechanisms before resistance becomes established.
